# Supplementary material for: Survival status and predictors of neonatal mortality among neonates admitted to Neonatal Intensive care Unit (NICU) of Wollega University referral hospital (WURH) and Nekemte Specialized hospital, Western Ethiopia: A prospective cohort study
Source: PLoS One. 2022 Jul 29;17(7):e0268744. doi: 10.1371/journal.pone.0268744 (PMC9337704; doi:10.1371/journal.pone.0268744)
Supplement: S2 File — (DOCX) [file pone.0268744.s002.docx]

# **Annexes**

## **Annex 1:- Data Extraction Tool**

**Introduction**

This patient information collection format was intended to assess **Survival status and predictors Neonatal mortality among neonates admitted to NICU in Western Ethiopia, Prospective Cohort study.** The study was conducted through interviewing mother and from medical cards of neonates. The study was aimed to fill the information gap and provide evidence for planners, decision makers and program implementers related to neonatal mortality in Ethiopia.

Date______/______ /_______ Questionnaire ID ___________

Name of data collector________________ signature_____

Name of supervisor ____________________Date__________ signature__________

Name of principal investigator ______________Date____________ signature_________

**Part I: Socio demographic Characteristics**

| Code | **Question** | **Response** | **Skip** |
| --- | --- | --- | --- |
| 101 | Age of mother | __________years |  |
| 102 | Residence where the mother came from | 1. Rural 2. Urban |  |
| 103 | Educational status of mother | 1. No education 2. Primary 3. Secondary 4. Tertiary and above |  |
| 104 | Employment status of mother | 1. Housewife 2. Government employee 3. Non-governmental employee 4. Daily laborer 5. Others(specify) |  |
| 105 | Marital status of women | 1. Never 2. Married 3. Divorced 4. Widowed |  |
| 106 | Religion of women | 1. Protestant 2. Orthodox 3. Muslim 4. Others(specify) |  |
| 107 | Ethnicity | 1. Oromo 2. Amhara 3. Gurage 4. Others (specify) |  |
| 108 | Wealth Index |  |  |

**Part II- maternal and neonatal history**

| 201 | Gestational age | 1. Preterm 2. Term 3. Post term |  |
| --- | --- | --- | --- |
| 202 | Birth weight | ____________gm |  |
| 203 | BMI | __________kg/m^2^ |  |
| 204 | Number of children | _________ |  |
| 205 | Number of pregnancy | _________ |  |
| 206 | ANC follow up | _______ |  |
| 207 | History of abortion | 1. No 2. Yes |  |
| 208 | Place of birth | 1. Home 2. Health institution |  |
| 209 | Mode of delivery | 1. Spontaneous vaginal delivery 2. operative (instrumental) vaginal delivery 3. cesarean section |  |
| 210 | Attendant of delivery | 1. TBA 2. Health professional |  |
| 211 | Type of delivery | 1. Single 2. Multiple |  |
| 212 | Preceding birth interval | ________yrs |  |
| 213 | Birth order | ________ |  |
| 214 | Presence of medical disease | 1. No 2. Yes | If No skip to 301 |
| 215 | If yes type of medical disease | 1. DM 2. HTN 3. Heart failure 4. Anemia 5. other(specify) |  |
| 216 | Distance of Health Facility | _______km |  |

**Part III-Immediate or interventional factors**

| 301 | Early initiation of BF | 1. No 2. Yes |  |
| --- | --- | --- | --- |
| 302 | Temperature | ______^o^c |  |
| 303 | Maternal complications | 1. No 2. Yes | If No skip to 305 |
| 304 | If yes types of Maternal complications | 1. Obstetric hemorrhage, 2. Puerperal sepsis 3. Prolonged labor, 4. Eclampsia and preeclampsia, 5. Mal-presentation and mal-position, 6. PROM, 7. Cord prolapse, 8. Obstructed labor 9. Cephalopelvic disproportion (CPD), 10. Emergency cesarean section, and 11. Retained placenta |  |
| 305 | Neonatal complications | 1. No 2. Yes | If No skip to 307 |
| 306 | If Yes types of Neonatal complications | 1. Asphyxia, 2. Prematurity, 3. Infection, 4. Jaundice 5. Others(specify) |  |
| 307 | Pregnancy complications | 1. No 2. Yes | If No skip to #309 |
|  |  |  |  |
| 308 | If Yes types of pregnancy complications | 1. Vaginal bleeding 2. Abdominal pain, 3. Persistence of back pain, 4. Blurry vision, 5. No fetal movement and swelling of hands or face |  |

**Part IV Follow Up information**

| 401 | Date of delivery | ______________ |  |
| --- | --- | --- | --- |
| 402 | Recent Date outcome occurred | _____________ |  |
| 403 | Age of neonate at the end of outcome | _____________days |  |
| 404 | Outcome at the end of follow up | 1. Death 2. Completed neonatal period 3. LTFU 4. On follow up when the study completed |  |
| 405 | Event at the end | 1. Censored 2. Death |  |
