# Supplementary material for: Survival status and predictors of neonatal mortality among neonates admitted to Neonatal Intensive care Unit (NICU) of Wollega University referral hospital (WURH) and Nekemte Specialized hospital, Western Ethiopia: A prospective cohort study
Source: PLoS One. 2022 Jul 29;17(7):e0268744. doi: 10.1371/journal.pone.0268744 (PMC9337704; doi:10.1371/journal.pone.0268744)
Supplement: Attachment — Submitted filename: response letter.docx [file pone.0268744.s003.docx]

Dear   Assistant Editor-in-Chief PLOS ONE

Dear Editor, this is regarding the entitled as “Survival status and predictors of neonatal mortality among neonates admitted to Neonatal Intensive Cara Unit (NICU) of Wollega University referral hospital (WURH) and Nekemte Specialized hospital, Western Ethiopia: a prospective cohort study” submitted to PLOS ONE. Thanks for your time and consideration in editing the manuscript. We have carefully read your comments and corrected inline of your comments and suggestions. All comments raised were edited and incorporated in the main manuscript. Some of the changes were highlighted with yellow color in the manuscript

When submitting your revision, we need you to address these additional requirements.

1. Please ensure that your manuscript meets PLOS ONE's style requirements, including those for file naming. The PLOS ONE style templates can be found at 
<https://journals.plos.org/plosone/s/file?id=wjVg/PLOSOne_formatting_sample_main_body.pdf> and 
<https://journals.plos.org/plosone/s/file?id=ba62/PLOSOne_formatting_sample_title_authors_affiliations.pdf>

**Response**: Thank you Dear editor, we accepted your comment. All the revision was made in line with the journal requirements including the figure.

2. Please include additional information regarding the survey or questionnaire used in the study and ensure that you have provided sufficient details that others could replicate the analyses. For instance, if you developed a questionnaire as part of this study and it is not under a copyright more restrictive than CC-BY, please include a copy, in both the original language and English, as Supporting Information. If the original language is written in non-Latin characters, for example Amharic, Chinese, or Korean, please use a file format that ensures these characters are visible.

**Response:** Dear editor, we have included questionnaire used in the study as a supplementary file

3. Please state whether you validated the questionnaire prior to testing on study participants. Please provide details regarding the validation group within the methods section.

**Response:** Thank you dear editor, the tool was not validated on study participants. However, to maintain the reliability and validity of the tool we have checked its consistency by translating from English language to local language, than retranslated back to ENGLISH by expert of the language.

4. Please amend your current ethics statement to address the following concerns: Please explain why written consent was not obtained, how you recorded/documented participant consent, and if the ethics committees/IRBs approved this consent procedure.

**Response**: Thank you dear editor. I know this very important question; however, written consent was not obtained from patient/care givers of the neonates. In Ethiopia, written consent is only possible if a sample is needed from the patients and any invasive procedure is performed. Since we were not received any blood sample and invasive procedure were not performed, only verbal consent was obtained from mothers.

5. Please ensure that you refer to Figure 3 and 7 in your text as, if accepted, production will need this reference to link the reader to the figure.

**Response**: revised

Additional Editor Comments:

I would like to commend the authors for their efforts in conducting this important study. the manuscript can be improved substantially with hard work. English language needs to be corrected, improved and tightened. Some improvements in the tables are required with clarity in the observed variables for example age to be written as age in completed years, distance in kilometers, the way the chronic medical diseases are given is confusing. categories should be very clear as no chronic condition, Hypertension, Diabetes or more than one comorbidity etc and abbreviations should be avoided. Discussion section is very weak and needs improvement in terms of explaining why babies breastfeeding were not initiated earlier may be mother was sick in ICU which we don't know or baby was premature and sick . The manuscript has the potential to be published but needs major revisions.Hypothermia is given as a cause of death, this needs good discussion as most of the babies who died in earlier ays must be hypothermic. Also data were collected from two hospitals but in discussion only one hospital is mentioned. In the discussion section lots of results are repeated which should be avoided.
**Response**: Thank you dear editor, we have answered your question in the discussion part of revised manuscript

[Note: HTML markup is below. Please do not edit.]

**Reviewers' comments:**

**Reviewer's Responses to Questions**

**Comments to the Author**

**Review Comments to the Author**

**Reviewer #1:** The present study entitled “Survival status and predictors of neonatal mortality among neonates admitted to Neonatal Intensive Cara Unit (NICU) of Wollega University referral hospital (WURH) and Nekemte Specialized hospital, Western Ethiopia: a prospective cohort study” by Tadesse Tolossa at al. is an interesting study with potential public health importance aimed to assess the survival status and predictors of neonatal mortality among neonates admitted to NICU of WURH and Nekemte Specialized Hospital, Western Ethiopia. However, following points should be addressed for strengthening the paper in order to maintain readers’ interest and to convey a clear message.

**Response**: Dear reviewer**,** thank you for taking a time to review our work thoroughly. We have tried to incorporate your comments in the main manuscript. In addition, here we have answered your questions. Hope we have addressed all your concerns

**Major revisions:**

• On page 5 lines 26-27, values for P1 and P2 considered for sample size calculation should be reported.

**Response**: Thank you Dear reviewer, we have accepted your comment and incorporated in the revised manuscript

• On page 6 line 1, does the total sample size (396) include lost to follow-up and/or those referred/transferred to other facility?

**Response**: Thank you Dear, actually 398 sample size was calculated by adding 10% non-response rate. It did not include lost to follow up and neonates referred for further treatment. They were included under censored data.

• On page 6 line 24, Birth weight categories (<2500gm, 2500 - 4000gm, and ≥ 4000gm) are not consistent with categories defined in Table 3 (<2500gm, 2500 – 3900, and ≥ 4000gm).

**Response**: Thank you dear, we have corrected it

• Did authors assess confounding and interaction? They should report it on page 8 under data management and analysis.

**Response:** Dear reviewer, thank you very much for this important question. Sorry, we have not considered interaction effect in our analysis, but we have controlled the effect of confounding by fitting multivariable analysis.

• Under Ethical Consideration on page 8, authors have mentioned that oral informed consent was obtained whereas about 80% mothers were literate. Was there any specific reason for not obtaining written informed consent? Please specify.

**Response**: Thank you dear editor. I know this very important question; however, written consent was not obtained from patient/care givers of the neonates. In Ethiopia, written consent is only possible if a sample is needed from the patients and any invasive procedure is performed. Since we were not received any blood sample and invasive procedure were not performed, only verbal consent was obtained from mothers.

• Result section can be improved further with more appropriate summary of results and by merging table 2 and Figure 1 to make one table of maternal obstetric characteristics and complications, Table 3 and Figure 2 for characteristics of newborn admitted to NICU and the reasons for admission, etc. Similarly, authors should also think about other figures.

**Response:** Thank you dear, we have merged figures ij table 2 and 3 as per your recommendations

• On page 13, line 12 (To see …………..technique was used.) and lines 16 – 19 (A separate graph of…………………..log rank test was computed.) are related to methods and can be moved there.

**Response**: Revised

• Similarly, on page 14, lines 15-16 (Covariates that had P- value ≤ 0.25…………………cox regression analysis) can also be moved methods section.

**Response**: Revised

• Table 4 presents multivariable model results with insignificant predictors (p>0.05). Authors are requested to revisit this multivariable analysis part to present final model results.

**Response:** Dear reviewer, we are very much grateful for your critical look into our manuscript. Regarding the model, we have raised a series of steps followed during analysis for building and selecting best fitting model in the previous reviewer’s comment. Firstly, we selected variables for multivariable analysis based on their bivariable P-value (0.25), secondly, we started to build model through backward selection method and thirdly, we selected the best model from the result of backward stepwise selection using log likelihood ratio (LLR). Here, we selected model one among the eight models built during the backward selection process because this model has relatively higher value of statistics. Obviously it is known that the higher the value of statistics, the better the model in Log likelihood ratio.

• Authors are advised to review list of reference again as some of the references seems incomplete (Refs 5, 9, 14, etc.)

**Response**: Revised

**Minor revision**

• Some language corrections will improve the overall quality of paper.

**Response**: Dear reviewer, we are thankful for your important comment and we have tried to edit the grammatical flaws throughout the manuscript in its revised version. We have edited the spelling, grammatical errors, incomplete and poorly structured sentences throughout the manuscript.

**Reviewer #2:** This is a critical area of study where data is either scarce or unavailable. This study methodology has several major flaws:

1. This study was carried out in two hospitals. I believe a hospital description, such as the number of beds in each ward and incubators or ventilators in the NICU, annual births, NICU admissions, mortality rate, and so on, would be beneficial. You also mentioned two other hospitals (Marie Stopes International and FGAE Hospital) - did these facilities take part in the study? If not, there is no need to mention it.

**Response**: Thank you dear reviewer, we have accepted your comment and included in the revised manuscript

2. Incomplete sample size assumptions (you should mention the percent of neonatal mortality among exposed and the percent of mortality among unexposed) Because there are many exposures, you must calculate sample size for each exposure and the highest sample size should be used for this study. Furthermore, you did not specify how much the sample size was inflated due to lost to follow-up/withdrawal from the study.

**Response**: Thank you dear reviewer, we have incorporated sample size issue in the revised manuscript

3. You must define variables as needed. Birth asphyxia, prematurity, sepsis, and so on.

**Response**: Thank you dear, we have accepted your comment and incorporated in the revised version of manuscript

4. "Data collection procedure" should be used instead of "Data collection tools and techniques."

**Response**: thank you dear, we have modified it

5. Please explain why oral consent was given.

**Response**: Thank you dear editor. I know this very important question; however, written consent was not obtained from patient/care givers of the neonates. In Ethiopia, written consent is only possible if a sample is needed from the patients and any invasive procedure is performed. Since we were not received any blood sample and invasive procedure were not performed, only verbal consent was obtained from mothers.

.

6. I suggest redo the analysis using standard categories. for example, term and preterm, ANC (no ANC vs. at least one ANC visit)

Response: Thank you dear, we have revised according to your comment

7. Figures 1 and 2 could be useful if categorized by baby outcome status (alive/dead).

**Response**: Dear reviewer, we believe your comment is worthwhile in improving the manuscript but we have removed figure 1 and 2 as per recommendation of reviewer 1.

8. Figure 4 is unnecessary.

**Response**: removed

9. What is the average length of survival?

**Response**: Thank you dear reviewer, the median survival time for overall neonates was 27 days and median time to death for event (death) was 10 days.

10. Please check for grammatical and punctuation errors and strongly advise having the manuscript reviewed by an English native speaker.

**Response**: Dear reviewer, we are thankful for your important comment and we have tried to edit the grammatical flaws throughout the manuscript in its revised version. We have edited the spelling, grammatical errors, incomplete and poorly structured sentences throughout the manuscript. Now we believe the revised version is clean and clear enough to the readers.
