# Supplementary material for: Survival status and predictors of neonatal mortality among neonates admitted to Neonatal Intensive care Unit (NICU) of Wollega University referral hospital (WURH) and Nekemte Specialized hospital, Western Ethiopia: A prospective cohort study
Source: PLoS One. 2022 Jul 29;17(7):e0268744. doi: 10.1371/journal.pone.0268744 (PMC9337704; doi:10.1371/journal.pone.0268744)
Supplement: Attachment — Submitted filename: response letter.docx [file pone.0268744.s004.docx]

Dear   Assistant Editor-in-Chief PLOS ONE

Dear Editor, this is regarding the entitled as “Survival status and predictors of neonatal mortality among neonates admitted to Neonatal Intensive Cara Unit (NICU) of Wollega University referral hospital (WURH) and Nekemte Specialized hospital, Western Ethiopia: a prospective cohort study” submitted to PLOS ONE. Thanks for your time and consideration in editing the manuscript. We have carefully read your comments and corrected inline of your comments and suggestions. All comments raised were edited and incorporated in the main manuscript. Some of the changes were highlighted with yellow color in the manuscript

**Editor comment**

In the abstract you mention: 'Studies are limited in Ethiopia in general, and no study has been conducted in the study area in particular'. However, we were able to find several studies on the topic:

**Response**: Thank you dear, we have accepted your comment and addressed in the revised manuscript. Yes a number of researches were conducted on this area, but the main gap is that the previous studies were limited to extracting data from patient cards, and only their focus was the survival status of neonates during the seven days of life or while they are in admission room. Only limited studies were conducted by following neonates for 28 days.
